# Two distinct light-induced reactions are needed to promote germination in spores of *Ceratopteris richardii*

**DOI:** 10.3389/fpls.2023.1150199

**Published:** 2023-06-02

**Authors:** Ashley E. Cannon, Tanya Sabharwal, Mari L. Salmi, Ganesh Kumar Chittari, Valli Annamalai, Lindsey Leggett, Hope Morris, Cameron Slife, Greg Clark, Stanley J. Roux

**Affiliations:** Department of Molecular Biosciences, University of Texas at Austin, Austin, TX, United States

**Keywords:** *Ceratopteris*, light signaling, phytochrome, chlorophyll, ATP, fern spore

## Abstract

Germination of *Ceratopteris richardii* spores is initiated by light and terminates 3-4 days later with the emergence of a rhizoid. Early studies documented that the photoreceptor for initiating this response is phytochrome. However, completion of germination requires additional light input. If no further light stimulus is given after phytochrome photoactivation, the spores do not germinate. Here we show that a crucial second light reaction is required, and its function is to activate and sustain photosynthesis. Even in the presence of light, blocking photosynthesis with DCMU after phytochrome photoactivation blocks germination. In addition, RT-PCR showed that transcripts for different phytochromes are expressed in spores in darkness, and the photoactivation of these phytochromes results in the increased transcription of messages encoding chlorophyll a/b binding proteins. The lack of chlorophyll-binding protein transcripts in unirradiated spores and their slow accumulation makes it unlikely that photosynthesis is required for the initial light reaction. This conclusion is supported by the observation that the transient presence of DCMU, only during the initial light reaction, had no effect on germination. Additionally, the [ATP] in *Ceratopteris richardii* spores increased coincidentally with the length of light treatment during germination. Overall, these results support the conclusion that two distinct light reactions are required for the germination of *Ceratopteris richardii* spores.

## 1 Introduction

Plants use many different environmental stimuli to regulate development and growth throughout their lifetime. The stimulus of light has an especially profound effect on plant development. Plants are sensitive to any illumination, sensing the quality, intensity, direction, and duration of the light and altering their development in relation to all of these factors. In seed plants, light responses occur throughout post-embryonic growth. Light affects seed germination, seedling development, shade avoidance, and the timing of flowering. In older plant lineages that reproduce using spores (e.g. ferns and mosses), light signaling is also of crucial importance, as revealed by several relatively recent studies (Li et al., 2015; Possart et al., 2017; Babla et al., 2019; Biswal and Panigrahi, 2020; Cai et al., 2021).

Phytochromes, photoreceptors primarily responsible for perceiving red and far-red light, are involved in initiating germination in seeds of flowering plants and in spores of ferns (Li et al., 2015; Rensing et al., 2016; Inoue et al., 2017; Legris et al., 2019). Phytochromes exist as two interconvertible conformations that have distinct absorption spectra. Pr, the inactive conformation, absorbs maximally in the red region (620 – 700nm, max = 668 nm), and Pfr, the active conformation, absorbs most strongly in the far-red region (700 – 850 nm, max = 730 nm). In the presence of red light, Pr is converted to Pfr; and far-red light converts Pfr back to Pr. Phytochrome can interact with other photoreceptor pathways and environmental sensing systems to regulate both photomorphogenesis (Franklin and Whitelam, 2004; Montgomery, 2016) and directional growth of organs, such as roots and shoots (Molas and Kiss, 2008; Vandenbrink et al., 2014; Sullivan et al., 2016; van Gelderen et al., 2018).

Light-induced germination has been extensively studied in the spores of different species (reviewed in Suo et al., 2015; Biswal and Panigrahi, 2020). In the ferns *Pteris vittata* and *Ceratopteris richardii* (hereafter referred to as simply *Ceratopteris*), which have photosynthetic gametophytes, red light (RL) induces and blue light (BL) inhibits spore germination (Sugai and Furuya, 1967; Cooke et al., 1987; Suo et al., 2015). However, in the club moss *Lycopodium clavatum*, which has non-photosynthetic gametophytes, far-red (FR) light and dark induces spore germination (Whittier, 2006; Whittier, 2008). The photoreversibility of these germination processes, either RL/FR or FR/RL, implicates phytochromes in the germination of spores with photosynthetic and non-photosynthetic gametophytes (Sugai and Furuya, 1967; Cooke et al., 1987; Whittier, 2008). In *Ceratopteris*, which has long been a favored system for studying growth and development of fern gametophytes (Chatterjee and Roux, 2000; Petlewski and Li, 2019), and which now has an annotated genome assembly (Marchant et al., 2022), it took 0.6 h of RL exposure for 50% of spores to escape FR induced photoreversibility (Cooke et al., 1987).

Light can also affect growth polarization in ferns in a differential growth response called phototropism. Some studies on the effects of light on growth direction in fern protonema, a filamentous structure present during early gametophyte development comprised of a tip-growing apical cell and other sub-apical, rod-shaped cells, have also implicated phytochrome as the primary photoreceptor responsible for this response (Wada and Kadota, 1989; Hayami et al., 1992; Kawai et al., 2003). In *Ceratopteris* spores, light affects spore polarization, leading to the rhizoid growing in the opposite direction of a unilateral light treatment (Edwards and Roux, 1998). In the fern, *Adiantum capillus-veneris*, the phototropic response is mediated by neochrome, a chimeric protein comprised of features characteristic of both phytochrome and the BL photoreceptor, phototropin (Nozue et al., 1998; Kawai et al., 2003; Kanegae and Kimura, 2015; Li et al., 2015). Neochrome originated in hornworts but was acquired by ferns through horizontal gene transfer (Li et al., 2014). The presence of this protein may explain why in some ferns, including *Adiantum capillus-veneris*, phototropism is controlled by both BL and RL. The presence of a chimeric photoreceptor and the ability to direct growth in response to both RL and BL point to the evolution of a complex light-sensing system. This system most likely evolved due to the low-level light environments that many older plant lineages experienced (Suetsugu and Wada, 2007; Cai et al., 2021).

Although light plays a role in directing the polarization of *Ceratopteris* spores, the direction of rhizoid growth upon spore germination is controlled primarily by gravity. When spores are grown in uniform light under stationary, 1*g* conditions, over 90% of rhizoids grow down. However, when orientation is continually changed on a rotating clinostat, the direction of rhizoid growth is random (Edwards and Roux, 1998). When spores are stationary, the direction of light can increase or decrease the effect of gravity-directed rhizoid growth. When light is bi-directional, both above and below germinating spores, the percentage of downward growing rhizoids is not significantly different from spores grown in uniform light. By contrast, when spores are grown in unidirectional light from above, parallel to the direction of gravity, gravity-directed polarization significantly increases. However, when spores are illuminated with unidirectional light from below, gravity-directed polarization is decreased by over 10% (Edwards and Roux, 1998). It is not surprising that *Ceratopteris* spores integrate both light and gravity during polarization, since this process is vital for survival.

The data and techniques described above motivated an effort to further clarify the role of light in controlling the polarization and subsequent direction of rhizoid emergence and growth during *Ceratopteris* spore germination. Light and the uptake of water initiate germination in *Ceratopteri*s spores. After 24-30 h of light exposure, the nucleus migrates downward based upon the direction of gravity. This is followed by the first cell division at 48-60 h after light exposure and the emergence of a polarly growing rhizoid at 72 h (Chatterjee and Roux, 2000). Because the polarization of spore cells by gravity is not fixed until at least 8 h after the initial light treatment initiates germination, but is definitively fixed by 48 h later (Edwards and Roux, 1994), we initially minimized the effects of light on the early polarized development of spores by keeping the cells in darkness between the first 8 and 48 h after germination initiation. Initial studies showed that when spores are exposed to 8 h of light, they can be moved to the dark and will still germinate if, and only if, they are re-illuminated after 48 h. Theoretically both phytochrome activation and photosynthesis could have been involved in spore responses to both the initial 8 h light treatment and the light given after 48 h. If only phytochrome activation was needed during the first 8 h of light, then much less light would be required, and inhibitors of photosynthesis would not negate the effects of this first light treatment. Moreover, learning when and why the second illumination was needed would help resolve whether both light responses were required for the completion of the germination process. Thus, investigating which light-driven processes were necessary to irreversibly induce germination in *Ceratopteris* spores would provide novel data and insight into the signaling responses that control spore germination, which are currently not well understood.

The preliminary data discussed above suggested the hypothesis that two separate light reactions were needed to irreversibly induce germination in *Ceratopteris* spores. Here we test this hypothesis. Additionally, we answer the questions of how much light is needed to permit spore development to progress to germination, and, when light is needed, whether the cellular mechanism needed for it to promote germination after 48 h is photosynthesis or another molecular process. To some extent, our studies parallel those of Murata and Sugai (2000). However, they reported on the effects of light on rhizoid emergence, not from spores, but from dark-grown prothallial cells. Their results implicated both phytochrome and photosynthesis in the development of rhizoids growing out from prothallial cells, but they did not define the light requirements for the earlier event of spore germination, defined as rhizoid emergence from the spore. Here we focus on the light requirements for spore development to progress all the way through to germination. Our study reports that two distinct light reactions promote *Ceratopteris* spore germination, one mediated by phytochrome to initiate spore germination, and the second mediated by chlorophyll to generate the photosynthetic energy needed for the differentiation of the rhizoid and its first emergence from the spore.

## 2 Materials and methods

### 2.1 Plant material and growth conditions

*Ceratopteris richardii* Brogn. spores were from an inbred diploid strain, Hn-n, kindly provided by Dr. Leslie G. Hickok. *Ceratopteris* spores were sterilized by soaking them in 20% (v/v) bleach with gentle agitation for 1.5 min then rinsing them three times with sterile water. The spores were then soaked in 10 mL of sterile water in the dark at 28°C for 5-7 days. After soaking, spores were rinsed three times with sterile water and ½-strength Murashige and Skoog (MS) medium (Caisson Labs), pH 6.3 with 1% (w/v) Bactoagar (Sigma Aldrich) was added in order to achieve a final spore density of 1 mg spores/mL of medium. Spores were sown in 65 mm x 15 mm petri dishes. Using a 1000 µL pipette, 1 mL of media with spores was added to each dish. The agar plates were left for 5 min to solidify before being wrapped in Parafilm and placed in a growth chamber. During spore sterilization and sowing, green light (550 nm) from a 25 W, incandescent bulb was used for illumination to eliminate any effects of white light exposure. Dishes were grown vertically in order to ensure that rhizoids grew down in a visual plane. The spores were grown in an incubator set at 28°C with illumination from either two 100W equivalent daylight (5000K, 400-800nm) LED bulbs located on one side of the chamber for white light or from a SNAP-LITE™ single beam, red light module positioned above the spores for red light (quantumdev.com, 670 nm, 10-14 PAR). Light was given either continuously throughout the germination period or for limited periods as defined. The fluence rate of the incident light was ca. 180 µmol m^-2^s^-1^. During dark treatments, spores were grown in a 28°C chamber that did not contain lights. Germinated spores were differentiated from nongerminated spores by the presence of a visible rhizoid. This criterion was used to distinguish germinated/non-germinated spores in all experiments presented in this manuscript. At least two technical replicates were used for each light treatment and each sample had three biological replicates with at least 100 spores each.

### 2.2 Inhibition of photosynthesis with DCMU

DCMU is a herbicide of the phenylurea class, and it inhibits photosynthesis by blocking the plastoquinone binding site of photosystem II (Hastings, 1960). Biochemical and genetic studies have demonstrated the specificity of this drug for photosynthesis, although in *Euglena* it can also delay the progression through the G2/M phase (Yee and Bartholomew, 1989). For DCMU treatments, spores were sown on microscope slides that had been lightly sanded on one side in order to prevent the spores and agar from slipping off. Spores were added to ½ strength MS (Caisson Labs), pH 6.3 with 1.25% (w/v) Bactoagar (Sigma Aldrich) in order to achieve a final density of 1 mg spores/mL of media. After the agar solidified for approximately 5–10 min, the slides were immersed in 20 mL of liquid ½ strength MS medium, pH 6.3 in a slide box. DCMU (Sigma-Aldrich), dissolved in ethanol, was added to the liquid media prior to pouring it into each slide box. The slides were maintained in 120 mg/L of DCMU for the limited period of time specified in the figure legends. This duration of DCMU treatment was standardly used in prior publications.

Reversal of the inhibitory effects of DCMU on photosynthesis was done by the washing method of Hastings (1960), who used this method to reverse the effects of DCMU treatment in the alga *Gonyaulax*. In short, the slides containing spores and agar were submerged in 20 mL of new liquid ½-strength MS, pH 6.3 without DCMU twice, for at least 1 h. Slides then remained in new liquid medium without treatment until visualization and assessment of germination at 96 h. Spores were considered germinated if at least one rhizoid that was at least one spore diameter long was present or if a prothallus was present. At least two technical replicates were used for each light treatment and each sample had three biological replicates with at least 100 spores each.

### 2.3 Measuring ATP and chlorophyll levels in spores exposed to various light conditions

For each sample, 10 mg of *Ceratopteris* spores were sterilized and soaked using the same protocol described previously. After the soaking, water was removed from the spores and replaced with 5 mL of ½ strength MS, pH 6.3 after which the spores were exposed to various light treatments, including: 96 h light, 1 h light/24-48 h light, 8 h light/48-49h light, 96 h light and DCMU treatment 0-24 h, 8 h light/48-96 h light and DCMU treatment, and 8 h light. After each light treatment, media was removed and the spores were rinsed once with 3 mL of ½ strength MS, pH 6.3, then 200 µL of ½ strength MS, pH 6.3 was added and spores were transferred to 1.5 mL tube and flash frozen in liquid nitrogen and stored in -80°C. In order to release intracellular ATP, the spore samples were ground to a powder using a pestle adapted to an electric motor and flash frozen again in liquid nitrogen. After all samples were powdered, the samples were thawed on ice and immediately boiled for 10 min. After boiling and cooling the samples, they were centrifuged at 17,000 *g* for 6 min at 4°C and 50 µL of the supernatant from each sample was added to a separate tube for quantification of ATP.

The dilutions for the ATP standard curve were made using ½ strength MS, pH 6.3 according to manufacturer’s protocol included in the ENLITEN^®^ ATP assay kit (Promega). During the luminescence assay, samples were used at a 1:500 dilution. Eight technical replicates were used for each light treatment and each sample had three biological replicates. Luminescence was measured using a 1 s exposure time with a Mithras Luminometer (Berthold Technologies).

For chlorophyll assays, 10 mg of *Ceratopteris* spores were sterilized and soaked using the same protocol described previously. After the soaking, water was removed from the spores and replaced with 5 mL of ½-strength MS, pH 6.3 after which the spores were exposed to various light treatments including: 96 h white light, 30 min white light or red light, 8 h white light or red light, and 0-8 h and 24-48 h white light and red light. After light exposure, spores were collected, and flash frozen in liquid nitrogen. Media was removed prior to adding 500 µL of 95% ethanol. Chlorophyll was extracted, measured, and quantified using a protocol previously described in Nayek et al., 2014.

### 2.4 Total RNA isolation and RT-PCR

**Figure 1:**
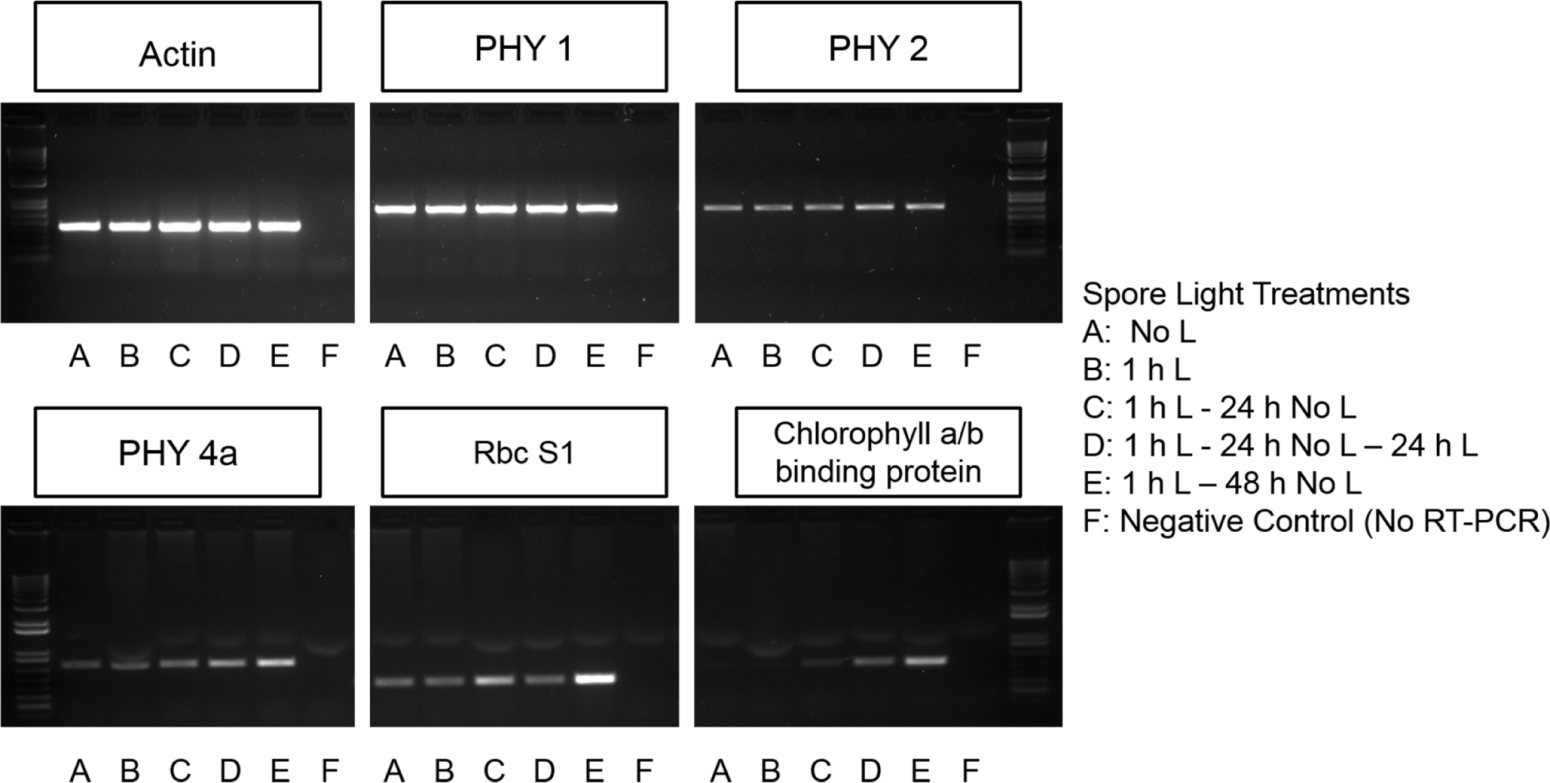
Chlorophyll a/b binding protein, Rubisco, and some phytochrome transcripts are light regulated. *PHYtochrome* 1 (*PHY1*) is present in spores prior to germination initiation and is not light dependent. *PHY2* is present prior to germination initiation and light exposure and time increases the abundance of this transcript. *PHY4a* and Ribulose bisphosphate carboxylase small chain 1(*RbcS1*) may be present prior to germination initiation but accumulation of *PHY4a* is also light and time dependent. Transcripts encoding chlorophyll a/b binding proteins do not begin accumulating until spores have been exposed to light.

RNA was isolated from spores using a protocol previously described in Salmi et al. (2005). However, rather than frozen plates of spores, 200 - 250 mg of spores were ground to homogeneity in a 1.5 mL tube with a pestle adapted to an electric motor. The purity and concentration of the RNA was determined using a Nano Drop UV-Vis spectrophotometer (ThermoFisher). The RNA was also checked for degradation by running a small volume of the extract on a 1% (w/v) agarose gel (Aranda et al., 2012). The cDNA synthesis was done using the SuperScript First Strand Synthesis System for RT-PCR (ThermoFisher). Primers specific to the *PHYtochrome* (*PHY*)*1, PHY2, PHY4a*, small subunit 1 of ribulose-1,5-bisphosphate carboxylase/oxygenase (*RbcS1*), dark-operative protochlorophyllide reductase subunit L (*ChlL*), light-dependent protochlorophyllide reductase (*POR*), and the chlorophyll a/b binding protein genes were used for a PCR using cDNA as a template. The *Ceratopteris* actin gene was used as positive controls in semi-quantitative RT-PCR (Primer sequences and melting temperatures can be found in **Supplementary Table 1**). Agarose gel electrophoresis (**Figure 1** and **Supplementary Figure 1**) was imaged using the Alphaimager Mini System with a 1.3-megapixel resolution camera. The AlphaView Stand Alone Analysis Software was used to acquire the .jpeg image files with a 2 sec exposure using auto contrast settings. No image manipulations or contrast enhancement has been done to these gel images. Two different gels are included in **Figure 1**, but the samples derive from the same experiment and the gels were run in parallel.

## 3 Results

### 3.1 *Ceratopteris* spore germination requires two light reactions

**Figure 2:**
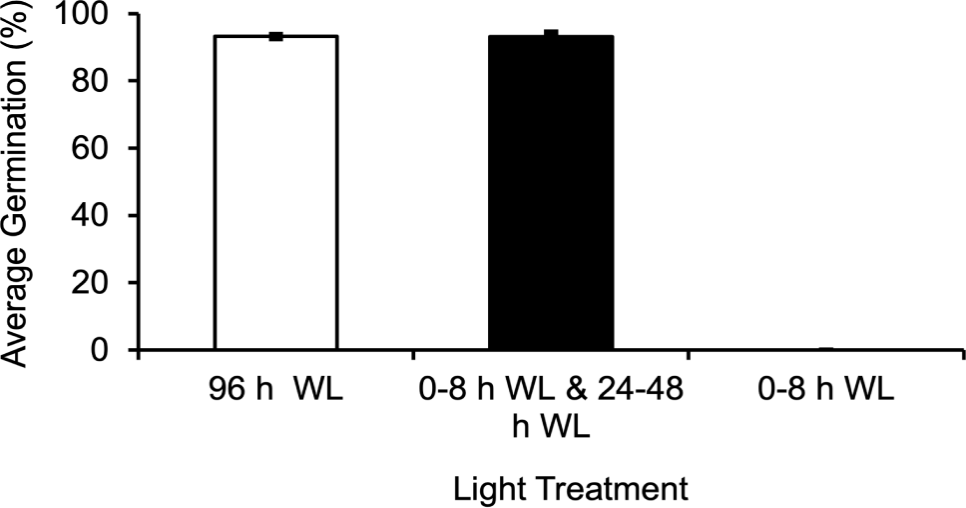
*Ceratopteris richardii* spores require two distinct light reactions in order to germinate. If spores are exposed to 8 h of WL (white light), moved to the dark until 48 h, and returned to the light until 96 h, the average germination rate is equivalent to that of spores grown in 96 h of light. If spores are only exposed to 8 h of light and placed in the dark until 96 h they will not germinate. Data shown is a mean ± SEM of six replicates and is representative of two independent experiments with at least 198 spores per replicate.

Initial experiments were carried out in which spores were exposed to 8 h of light (5000K, 400nm – 750nm, fluence rate = 180 µmol m^-2^s^-1^) and then moved to darkness until 48 h. If the spores were continuously irradiated between 48 and 96 h, the germination percentage, defined as the percentage of spores in which the spore coat has split and the first rhizoid has emerged, was the same (p-value=1.0) as if spores were continuously irradiated throughout the 96 h period (**Figure 2**). On the other hand, if the spores were not exposed to any additional light after the initial 8 h irradiation, then spores did not germinate even 5 d later (**Figure 2**). This result indicated a second light stimulus was needed for spore development to progress all the way to germination.

**Figure 3:**
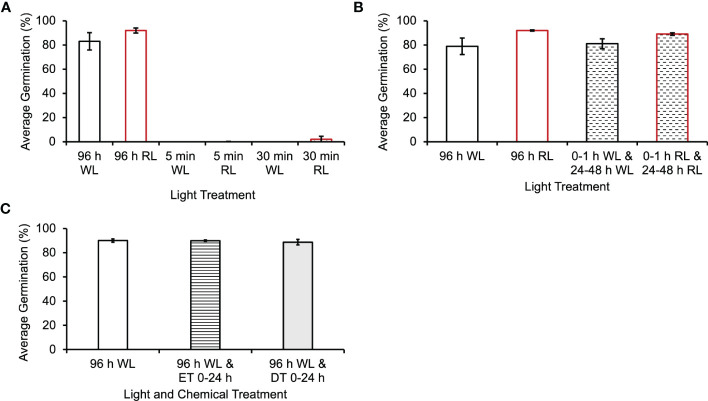
*Ceratopteris richardii* spores do not require photosynthesis for germination initiation. **(A)** Spores exposed to 5 min or 30 min of WL (white light) or RL (red light) do not germinate or germinate statistically significantly less than spores exposed to 96 h of WL or RL. **(B)** Spores exposed to 1 h of WL or RL, placed in the dark until 24 h and placed back in the light from 24-48 h, germinate at the same rate as spores exposed to 96 h of continuous light. **(C)** Inhibiting photosynthesis with DCMU during the first 24 h of development does not decrease the germination rate. Error bars represent standard error of the mean. Ethanol Treatment (ET) was 0.02% (v/v), the same solvent used for the DCMU Treatment (DT). Data shown is a mean ± SEM of six (A and B) or 9 **(C)** replicates and is representative of two (A and B) or three **(C)** independent experiments with at least 100 spores per replicate.

**Figure 4:**
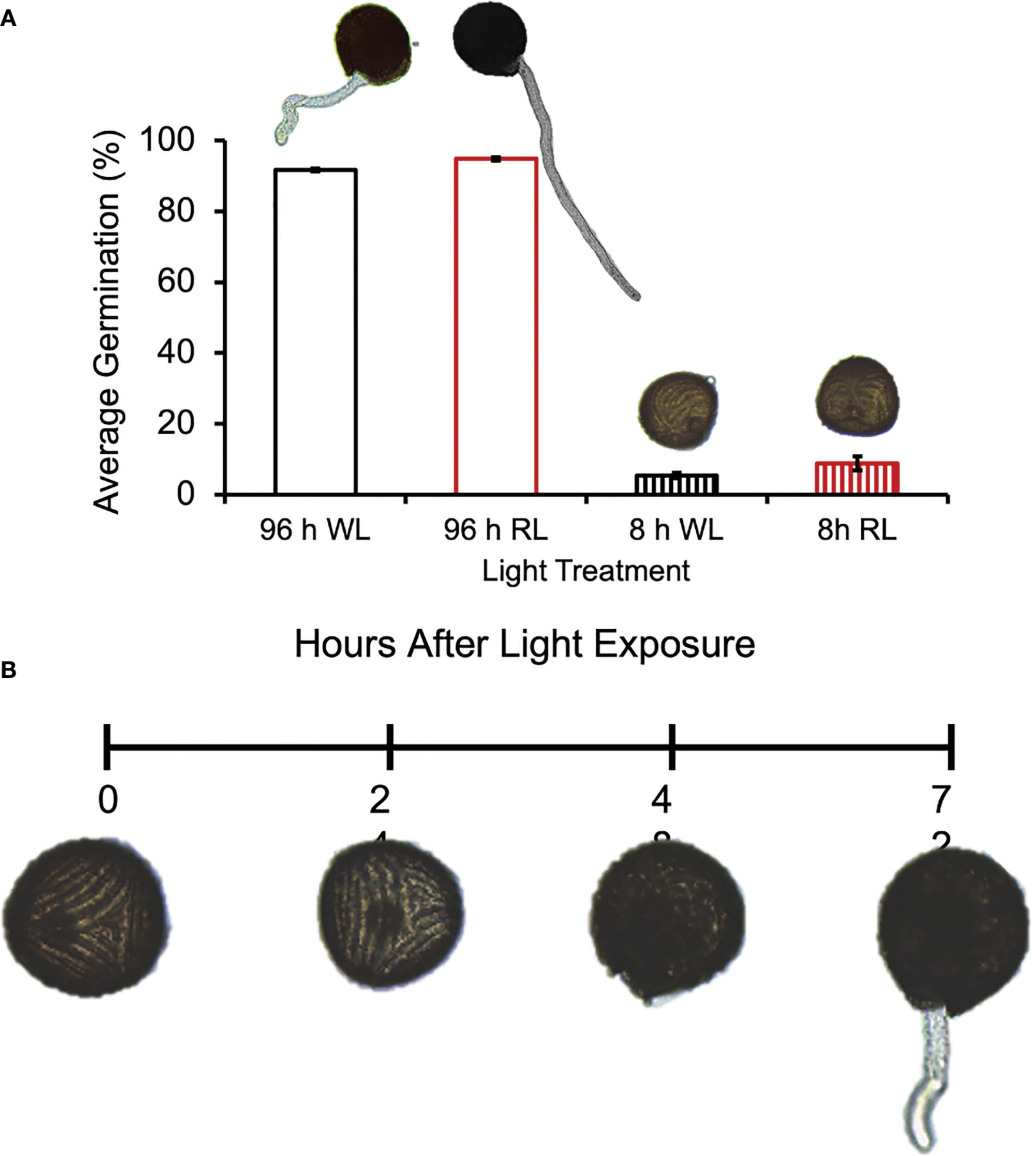
*Ceratopteris richardii* spores require more than 8 h of light to germinate. **(A)** Spores exposed to 8 h of WL (white light) or RL (red light) do not germinate or germinate statistically significantly less than spores exposed to 96 h of WL or RL. Spores show no visible differences after 8 h of WL or RL exposure. The mean difference between 96 h WL and 8 h WL was 86.3% and the mean difference between 96 h RL and 8 h RL was 85.9%. **(B)** Timeline of Ceratopteris spore development from 0 – 72 h of light exposure. After 48 h of light exposure, Ceratopteris spores showed visible signs of cell division and expansion. These visible changes included darkening of the spore coat, opening of the spore coat at the trilete, and visible emergence and growth of the rhizoid after 72 h of light exposure. Data shown is a mean ± SEM of three replicates and is representative of three independent experiments with at least 100 spores per replicate.

The 8 h of light given in the initial experiments would theoretically suffice to both activate phytochrome and initiate photosynthesis. To decrease the probability that photosynthesis was playing a major role during the initial light period, this light exposure was decreased to 1 h or less. In addition, since previous studies have shown that spores from *Pteris vittata* and *Adiantum capillusveneris* ferns germinated after a single short period of high-intensity, RL (650-670nm) irradiation (Sugai and Furuya, 1967; Sugai and Furuya, 1985), we treated *Ceratopteris* spores with relatively short periods of white light (WL) and RL. **Figure 3A** shows that 5 min or 30 min of WL or RL was not sufficient to induce germination in *Ceratopteris* spores. However, **Figure 3B** shows that 1 h of initial WL or RL was enough to initiate germination as long as the spores were placed back in the light from 24 - 48 h. After these light treatments, spores germinated equivalently to spores exposed to 96 h of light. A oneway ANOVA showed that there was no statistical difference between the four light treatments (p-value=0.2). To further test whether photosynthesis was playing a role during the first 24 h of irradiation, the spores were treated with the photosynthesis inhibitor, 3-(3,4-Dichlorophenyl)-1,1Dimethylurea (DCMU), during this period to inhibit photosynthesis, but then the inhibitor was washed out and the spores remained in the light for the remaining 72 h. These results showed that under these conditions, germination was still initiated, and spores germinated as though they had been exposed to 96 h of light without the inhibitor (**Figure 3C**), giving strong evidence that the inhibitor was effectively washed out. A one-way ANOVA showed that there was no statistical difference between the light and chemical treatments (p-value=0.8). To determine what cellular events happen after 8 h of light exposure, *Ceratopteris* spores were exposed to 8 h of WL or RL and moved to the dark until observation at 96 h after light exposure. Like the results shown in **Figure 3A**, 8 h of WL or RL was not enough to induce germination in *Ceratopteris* spores (**Figure 4A**). Spores exposed to 8 h of WL or RL look similar to spores exposed to 0 – 24 h of white light (**Figures 4A, B**). After 48 – 72 h, *Ceratopteris* spores showed visible signs of cell division and expansion (**Figure 4B**). These visible changes included darkening of the spore coat, opening of the spore coat at the trilete, and visible emergence and growth of the rhizoid.

**Figure 5:**
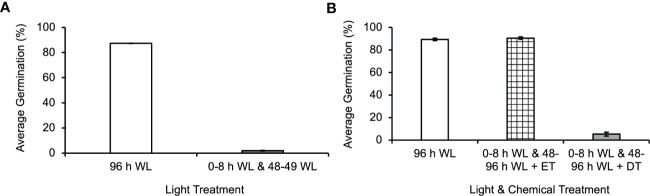
Photosynthesis is required for germination to continue through energy intensive developmental events in *Ceratopteris richardii* spores. **(A)** The second light treatment must be longer than 1 h in order for germination to progress. Statistical analyses (see Results section) indicate that a second light treatment of only 1 h resulted in a statistically significant decrease in germination. The mean difference between 96 h WL and 0-8 h WL and 48–49 WL was 85.3%. **(B)** Inhibiting photosynthesis during the second L (Light) treatment, 48 - 96 h, prevents germination. The second light treatment included the addition of 0.02% (v/v) ethanol treatment (ET) as control for comparison with DCMU treatment (DT). *Statistical analyses (see Results section) indicate that DCMU caused a statistically significant decrease in germination. The mean difference between 96 h WL and 0-8 h WL and 48-96 h WL + DT was 84% and the mean difference between 0–8 h WL and 48–96 h WL + ET and 0-8 h WL and 48-96 h WL + DT was 85.1%. Data shown is a mean ± SEM of 12 - 15 replicates and is representative of four independent experiments with at least 100 spores per replicate.

In flowering plants, the photoactivated form of phytochrome (Pfr) undergoes a reversion back to the inactive Pr form in darkness, so it was theoretically possible that the second light treatment was needed only to return phytochrome to its active Pfr form. To test this hypothesis, the second light treatment at 48 h was reduced to 1 h, a length of light period sufficient to activate phytochrome (Cooke et al., 1987), but not sufficient to generate much photosynthetic activity. Spores exposed to 8 h of initial light, and 1 h of light between 48 and 49 h had a germination rate of only 2% (**Figure 5A**). A one-way ANOVA (p-value=2.7x10^-9^) showed that there was a difference among the light treatments, and *post-hoc* analysis using the Tukey-Kramer test showed that a 1 h light exposure caused a statistically significant decrease in germination (p-value=1x10^-3^). The mean difference between 96 h WL and 0-8 h WL and 48 – 49 WL was 85.3%. These results are consistent with the hypothesis that the second light treatment has to be long enough to provide sufficient photosynthetic energy for the spore to proceed through germination and that the critical photoreceptor for the second light treatment is chlorophyll.

Spores were exposed to 8 h of initial light (5000K, 400nm–750nm, fluence rate = 180 µmol m^2^s^-1^), and then DCMU was added to the spores prior to returning them to the light from 48-96 h in order to determine if photosynthesis was required during the second light exposure. DCMU inhibits photosynthesis by disrupting the electron transport chain. When photosynthesis was inhibited during the second light treatment, an average of 5% of spores germinated (**Figure 5B**). A one-way ANOVA (p-value=1.1x10^-16^) showed that there was a difference among the light and DCMU treatment. Posthoc analysis using the Tukey-Kramer test showed that DCMU caused a statistically significant decrease in germination (p-value =1x10^-3^). The mean difference between 96 h WL and 0-8 h WL and 48-96 h WL + DT was 84% and the mean difference between 0–8 h WL and 48–96 h WL + ET and 0-8 h WL and 48-96 h WL + DT was 85.1%. The low percentage of spores that germinate after this light and DCMU treatment could indicate that a few of the spores in the population assayed had received enough light during the initial 8 h to generate the energy necessary for the remainder of germination. Alternatively, this result could be evidence that the concentration of DCMU used was not high enough to fully inhibit photosynthesis in all of the spores.

### 3.2 Chlorophyll a/b binding protein, Rubisco, and some phytochrome transcripts are light regulated

The genetic components expected to be involved in the initial light reaction were investigated using RT-PCR. RNA was extracted from *Ceratopteris* spores after various light treatments to determine if transcripts encoding phytochrome (*PHY*), ribulose-1,5-bisphosphate carboxylase/oxygenase (*RbcS1*), and chlorophyll a/b-binding proteins are expressed in spores. Results in **Figure 1** shows that the transcription of mRNA for chlorophyll a/b binding protein is initiated by light exposure and requires over 1 h to accumulate. This expression pattern makes it unlikely that photosynthesis is required for the first light reaction. However, *PHY1*, *PHY2*, *PHY4a* and *RbcS1* transcripts are present in spores that have never been exposed to light and the relative abundance of *PHY2*, *PHY4a*, and *RbcS1* transcripts increases over time in the dark after an initial 1 h light exposure (**Figure 1**). The transcript levels of dark-operative protochlorophyllide reductase (DPOR) and light-dependent protochlorophyllide reductase (POR) were examined in spores. Although there was a detectable level of DPOR transcript in spores independent of light treatment, there was no expression of POR (**Supplementary Figure 2**).

The differential accumulation of *PHY* transcripts shows that the expression of *PHY2* and *PHY4a* in *Ceratopteris* is light dependent. It also reveals that it takes 1 h or less than 1 h of light to initiate the expression of *PHY2* and *PHY4a* transcripts and that the level of *PHY1* transcripts is stable in spores in both darkness and light. Overall, **Figure 1** provides more support for the hypothesis that phytochrome may be a photoreceptor needed for the first light reaction in *Ceratopteris* spores. In addition, these results show that 1 h of light initiates the synthesis of transcripts for chlorophyll a/b binding proteins, but it takes 24 h before these transcripts begin to accumulate. Although these data are consistent with prior reports showing that phytochrome induces spore germination in *Ceratopteris* (Cooke et al., 1987; Possart and Hiltbrunner, 2013) and phytochrome induces the expression of chlorophyll a/b binding proteins (Silverthorne and Tobin, 1984), definitive evidence of which phytochrome was involved would require genetic knockouts, which is beyond the scope of this report. The transcript levels of DPOR and POR transcripts point to the light-independent synthesis of chlorophyll which is further supported by results shown in **Supplementary Figure 3** indicating that chlorophyll is detectable in spores after minimal light treatment.

### 3.3 ATP levels increase coincidentally with the length of light treatment

**Figure 6:**
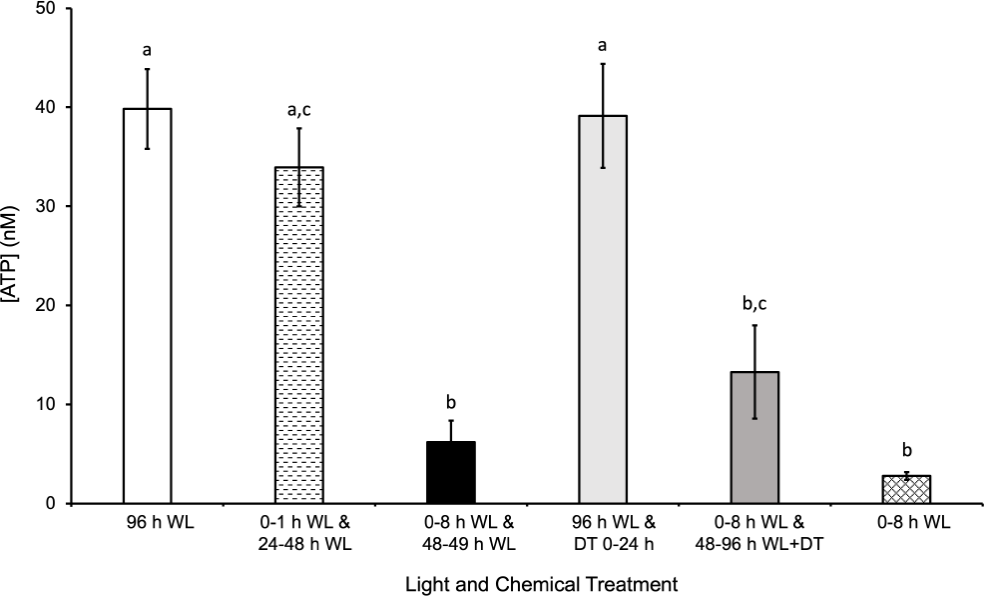
Effects of light on quantity of ATP extracted from spores. After 96 h of WL (white light), there was significantly more ATP extracted from spores relative to 0-8 h WL and 48-49 h WL, 0-8h WL and 48-96 h WL + DT (DCMU treatment), and 0-8 h WL. The mean difference between 96 h WL and 0-8 h WL and 48-49 h WL was 34 nM ATP. The mean difference between 96 h WL and 0-8 h WL and 48-96 h WL + DT (DCMU treatment) was 27 nM ATP. The mean difference between 96 h WL and 0-8 h WL was 37 nM. The quantity of ATP extracted from spores exposed to 0-1 h WL and 24-28 h WL and 96 h WL and DT 0 – 24 h was not significantly different from 96 h of WL. The mean difference between 96 h WL and 0-1 h WL and 24-48 WL was 6 nM. The mean difference between 96 h WL and 0-8 h WL was 37 nM. The highest [ATP] observed is coincident with light treatments and germinate rates. Data shown is a mean ± SEM of three replicates and is representative of two independent experiments. Letters above the bars represent statistical differences between the conditions. For all conditions with the same letter, the difference in ATP quantity is not statistically significant. If two conditions have different letters, then the quantity of ATP is statistically different.

In order to determine how a major product of photosynthesis, ATP, was affected during various light treatments, a luminescence assay was used to determine the [ATP] in spores after they were exposed to each treatment. As shown in **Figure 6** and **Supplementary Figure 4**, there was a significantly higher [ATP] after 96 h of continuous WL relative to a shorter WL treatment of 8 h (pvalue=4x10^-3^). The mean difference between 96 h WL and 0-8 h WL was 37 nM. Even though 8 h of light is sufficient to induce the production of chlorophyll and other components of the photosynthetic apparatus, the ATP generated by this light is apparently insufficient to support the completion of the developmental steps leading to germination. However, there was no significant difference in [ATP] after a 1 h and 12 h white light treatment at 0 h and 24 h after sowing, respectively, and 96 h of white light (p-value=0.9). The mean difference between 96 h WL and 0-1 h WL and 24-48 WL was 6 nM. The additional light treatment from 24-48 h was sufficient to provide the energy necessary to produce relatively similar levels of ATP as 96 h of light.

The [ATP] measured after the different light treatments tested would be the equilibrium level of ATP maintained in a cell as a result of the production and turnover of ATP during regular cellular processes. The decrease in ATP levels after a WL treatment of 8 h and 1 h at 0 h and 48 h, respectively, indicates that the spore is utilizing ATP for various processes and is unable to replenish it in the dark. The highest [ATP] observed is coincident with light treatment and would be expected to be a result of increased photosynthetic activity and does not happen without light treatment. This light effect is likely to be in part due to increased photosynthetic activity, although there are other metabolic processes that could contribute to the increased ATP level. The role of photosynthetic activity in ATP production during the second light treatment is supported by the statistically significant decrease in [ATP] when spores are treated with DCMU while being exposed to light from 48-96 h during a 96 h time period. In addition, the insignificant difference between spores exposed to 96 h light and spores exposed to 96 h light with DCMU from 0 – 24 h provides further support for this hypothesis.

In addition to the [ATP], we also measured the amount of chlorophyll accumulation after various light treatments and found the even in spores exposed to 30 minutes of light, there was a measurable amount of chlorophyll (**Supplementary Figure 3**). As the length of light treatment increase, the amount of chlorophyll accumulation increased. Overall, the results shown in **Figure 6** and **Supplementary Figure 3** support the conclusion that ATP is being consumed by energy-driven processes taking place in the cell during spore development, and unless this ATP is replenished and maintained at a high enough equilibrium level by photosynthesis, the spores cannot complete the germination process. Furthermore, the results also support the conclusion that although chlorophyll is synthesized during early development of *Ceratopteris* spores, the duration of light exposure must be long enough to provide the energy necessary for germination.

## 4 Discussion

During these experiments, it was determined that *Ceratopteris* spores must be returned to the light after a short, initial exposure in order to complete germination. The data in **Figures 2** and **3** show that less than 8 hours of light is required in order to initiate spore germination, but a second and longer light treatment is necessary to complete this process. The first light treatment needed to initiate germination is *Ceratopteris* spores can be as short as 1 h in length. As shown in **Figures 3** **–** **5**, the first light reaction is needed to initiate spore germination and is mediated by phytochrome (Cooke et al., 1987) and does not require photosynthesis. However, phytochrome photoactivation during the second light period does not suffice to support spore development through germination.

Regarding gene expression changes induced by phytochrome that would help promote spore germination, here we documented the light-induced upregulation of RUBISCO and chlorophyll-a/b binding proteins, which are needed in large amounts for photosynthesis (**Figure 1**). However, there is no doubt that light induces many other gene expression changes that could help mediate the spore germination process. Some of these were listed among the tentative unique genes (TUGs) documented by Salmi et al. (2005) to be significantly more abundant when spores were initially exposed to light (0 h) relative to 48 h later, and which would also support photosynthesis. For example, transcripts from TUGs associated with light-regulated chloroplast and/or photosynthesis processes, such as one encoding a putative peptidyl-prolyl cis-trans isomerase (BE641059), a photochlorophyllide reductase subunit *ChlL* (BE640829), and a photosystem-1 F subunit precursor (BE640963), were more abundant when spores were initially exposed to light (Salmi et al., 2005). Additionally, transcripts from TUGS that have sequence similarity to signal transduction pathway components that could be light- and/or phytochrome-activated and involved in the process of emergence from dormancy were also more abundant in spores after the initial light exposure. For example, the TUG BE641708 has significant sequence similarity to *COP1*, which is known to play a role in the photomorphogenesis signal transduction pathway (Wei et al., 1994). Also, G-protein signaling has been implicated in phytochrome and germination responses (Assmann, 2005; Chen, 2008; Urano et al., 2013), and, quickly after light exposure, transcripts from a TUG similar to a large GTP-binding protein (BQ087312), and transcripts from a TUG similar to a G-protein coupled receptor (BE642212) were significantly more abundant. Collectively, these gene expression changes during germination initiation in *Ceratopteris* spores suggest that during the first light reaction in Ceratopteris spores, phytochrome could mediate the process of emergence from dormancy using some of the same molecular components previously identified in flowering plants. Interestingly, a recent study by Marchant et al. provided transcriptome data from immature *Ceratopteris* gametophytes that complements and expands upon the data that was collected in germinating spores. The most abundant transcripts (3 TPM or more) in immature *Ceratopteris* gametophytes are enriched in protein domains for chlorophyll a-b binding protein and ribulose bisphosphate carboxylase small chain. Furthermore, that top four most highly enriched GO terms are all related to photosynthesis and generation of energy (GO:0015979, GO:0009765, GO:0019684, GO:0006091). The enrichment of these domains and GO terms points to the critical importance of the induction and expression of photosynthesis related genes in the early development of *Ceratopteris*.

In addition, these results suggest that the reason the second light treatment must be longer than 1 h is that the critical process supported by the second light treatment is photosynthesis. Of course, the second light treatment given at 48 h would re-activate any phytochrome that had undergone dark reversion to the inactive Pr form. Because the Pfr form of phytochrome induces cell division in dark-grown *Ceratopteris* prothalli (Murata and Sugai, 2000), it is possible that the second light treatment is required not only to generate photosynthetic energy but also to induce phytochrome-regulated signaling steps and gene expression changes needed for the first cell division at approximately 60 h after light exposure and for the emergence of the rhizoid at approximately 72 h after light exposure (Chatterjee and Roux, 2000).

Previous studies have shown that the second light treatment may only be necessary for growth and differentiation of the rhizoid initial rather than cell division. Yatsuhashi et al. (1996) showed that two distinct phytochrome responses are necessary for germination and the growth of rhizoids in *Dryopteris filix-mas* spores, the first one was required for the formation of chlorophyll and celldivision, while the second one was needed to promote growth and differentiation of the rhizoid initial. The results reported here for *Ceratopteris* do not resolve whether the second light treatment is required to both photoactivate phytochrome and generate photosynthetic energy, or whether its role is for photosynthesis only. These data also do not distinguish which cellular events require the second light treatment to progress (**Figures 4** and **5**). The lack of chlorophyll a/b binding protein transcripts in dark-incubated spores (**Figure 1**) supports the conclusion that an initial light treatment is necessary for the formation of chlorophyll. However, the photoreceptors and light treatments needed to induce the first cell division are still unknown.

The need for a second light reaction and the role of photosynthesis during this light reaction can be further explained by the developmental requirements of fern spores. In contrast to seeds, fern spores contain a single cell that must divide and give rise to additional cells that will differentiate into the rhizoid or the initial prothallium cell. Previous studies have shown that phytochrome induces the first cell division in *Adiantum capillus-veneris* spores after 5 min of red light treatment (Furuya et al., 1997). Similarly, *Pteris vittata* spores can be induced to germinate with as little as 2 minutes of highintensity, red light (Sugai and Furuya, 1967). These results suggest that a brief, red light exposure can induce the first cell division in some fern spores. In contrast, our results suggest that *Ceratopteris* spores require more light in order for their development to progress all the way through to germination. After light and water absorption, the energy for development is initially generated from respiration using stored energy reserves, but photosynthesis is required for the emergence and growth of the rhizoid. This conclusion is further supported by results from the ATP and chlorophyll assays shown in **Figure 6** and **Supplementary Figure 3**. ATP, a major product of photosynthesis, required more than 1 h of light in order to begin accumulating. The [ATP] increased above equilibrium levels when spores remained in the light during germination. Collectively, the germination and ATP assays show that the [ATP] present after one light treatment for 1 h or 8 h is not enough to complete germination. However, after a 1 h and 24 h light exposure at 0 h and 24 h after spore sowing, respectively, the level of ATP was not significantly different than 96 h of light, further supporting the conclusion that photosynthesis is required in order for *Ceratopteris* spores to complete germination.

Overall, these results confirm that two distinct light reactions are needed to promote germination in *Ceratopteris* spores, and they point to an evolutionarily conserved mechanism of light-regulated germination in plant systems. They also show that in order to ensure survival, *Ceratopteris* spores have developed a mechanism to stall development temporarily if light is scarce during energy intensive processes. Although this study provided some insight into the process of spore germination in *Ceratopteris*, to fully understand the role of photosynthesis in spore germination, future experiments should determine the rate of ATP depletion in light-activated spores and when photosynthesis becomes the primary source of energy. In addition to this study, other recent studies have also provided insights into the evolution and development of land plants and pointed to unique processes dictating the development of fern gametophytes. Scientists recently studied the mechanisms of meristem development in two fern systems, *Ceratopteris* and *P. vittata* (Wu et al., 2021). In both fern systems, patterns of cell division during gametophyte development led to specific cell identities and as a result, led to specific meristem behaviors. This developmental characteristic is different than studies in *Arabidopsis* where patterns of cell division in meristems are not linked to cell fate. Comparing the developmental processes in *Ceratopteris* to other land plants will expand our understanding of land plant evolution. Finally, future studies in *Ceratopteris* can now include genome-enabled research due to the recently published chromosomal genome assembly (Marchant et al., 2022).

## Data availability statement

The raw data supporting the conclusions of this article will be made available by the authors, without undue reservation.

## Author contributions

AC, MS, and SR conceived and designed research. AC, TS, MS, GK, VA, LL, HM, and CS conducted experiments and analyzed data. GC provided critical laboratory and grant support for the experiments. AC, TS, and SR wrote the manuscript. All authors contributed to the article and approved the submitted version.
