# Supplementary material for: Two distinct light-induced reactions are needed to promote germination in spores of Ceratopteris richardii
Source: Front Plant Sci. 2023 Jun 2;14:1150199. doi: 10.3389/fpls.2023.1150199 (PMC10272463; doi:10.3389/fpls.2023.1150199)
Supplement: Supplementary file 1 [file DataSheet_1.pdf]

Gene	Sequence	Tm
C. richardii Actin Forward	GAA AAT GGC TGA TGT GGA CGA AG	56.5
C. richardii Actin Reverse.	GCA TAA AGT GAT AAC ACA GCT TG	52.4
C. richardii RbcS1 Forward	GGA AGT CTC TTG ATG CCA GCT	57.1
C. richardii RbcS1 Reverse	TCG TGT ATT GTC GAA GGC CAA	56.6
C. richardii PHY1 Forward	AAA GCA TAG GAA ACG GAG CGG	57.8
C. richardii PHY1 Reverse	CCA TTT TGG CTG TTG ATG CGG	57.6
C. richardii PHY2 Forward.	TCT GGC AGC TTG GAG TCA CT	58.6
C. richardii PHY2 Reverse	GCA ATG CCA GAT CCA GCA CT	58.3
C. richardii PHY4A Forward	TGG CTC CTG GAT GTG CAT AGA	58.2
C. richardii PHY4A Reverse	GGA CCA TTC TGA GCC TGT TCG	58.0
C. richardii Chlorophyll a/b Binding Protein Forward	ATG GTC GAT TCT GGA CTT GCT	61.0
C. richardii Chlorophyll a/b Binding Protein Reverse	GGC AAA GAG ATC TGC ACA GG	60.9
C. richardii ChlL Forward	ACG GAA AAG GCG GAA TTG GA	57.3
C. richardii ChlL Reverse	TTC TCG AAC CGA AGC GGA AA	56.8
C. richardii POR Forward	GCA GCA CCA GTG GAG ACA AT	57.9
C. richardii POR Reverse	TCT CCT GAG TCA ACC AAC GC	57.2

Supplemental Table 1: Sequence and melting temperature of primers used for RT-PCR

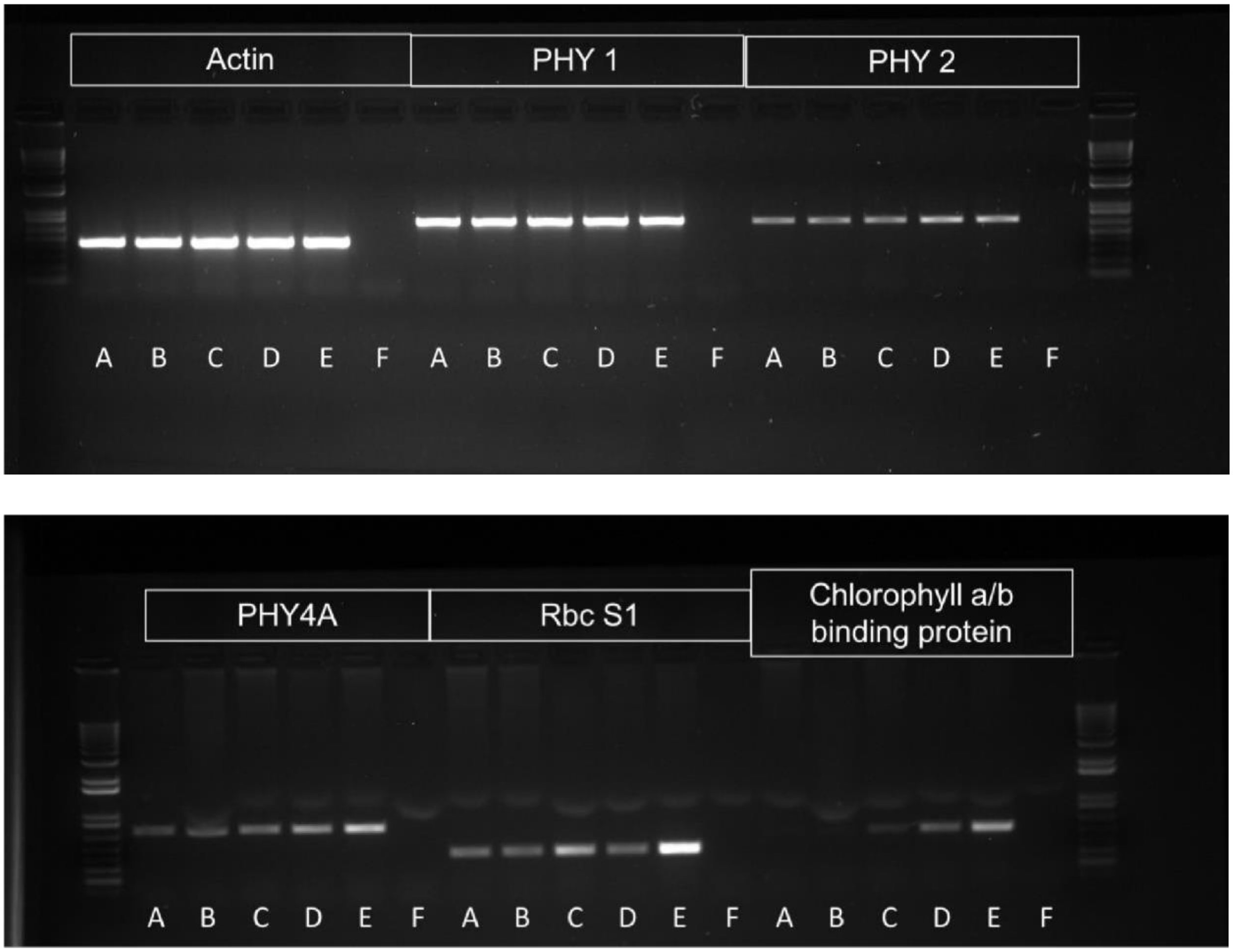

Supplemental Figure 1: Two original gel images used to create Figure 5. The annotations used to label the images above correspond to the same annotations used in Figure 5.

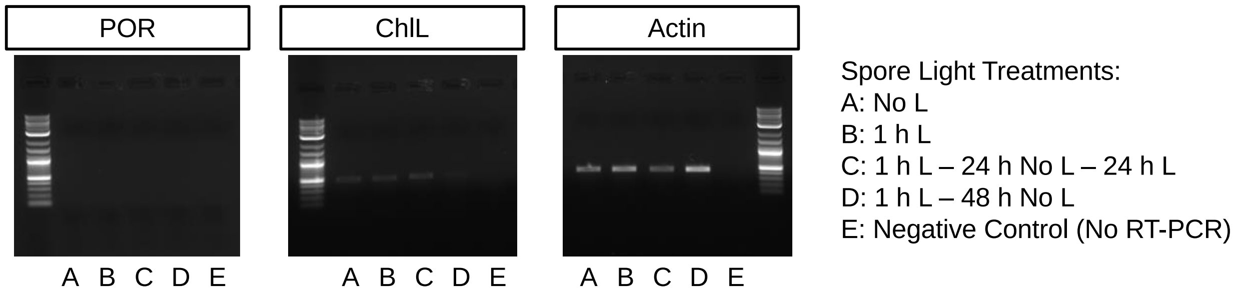

Supplemental Figure 2: Effects of light treatments on *POR* and *ChlL* transcripts.

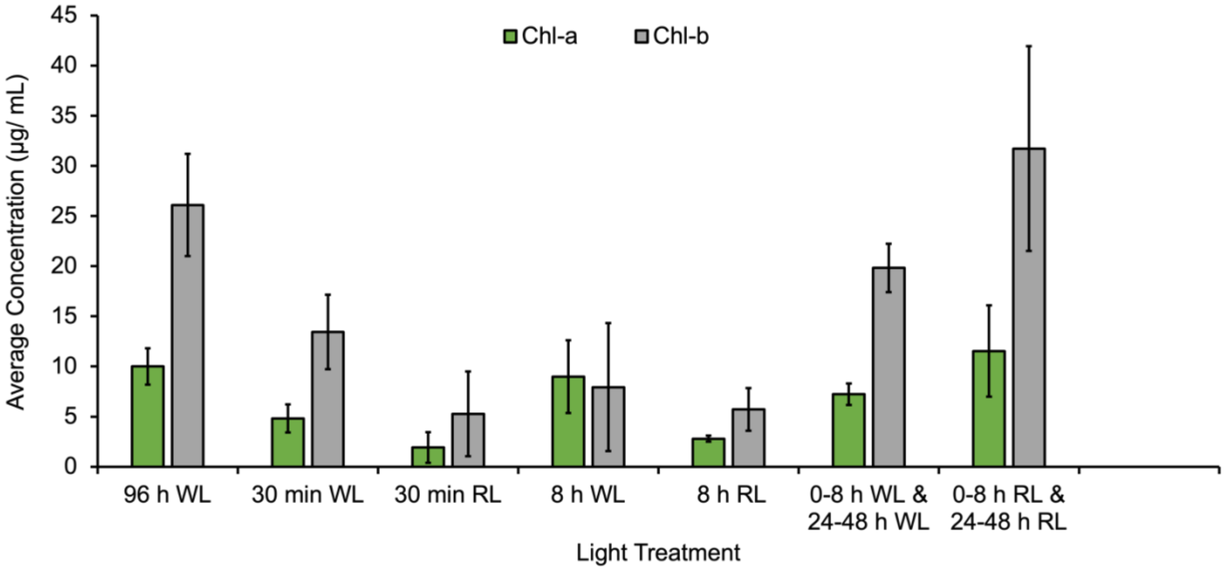

Supplemental Figure 3: Concentration of chlorophyll in *Ceratopteris* spores after various light treatments.

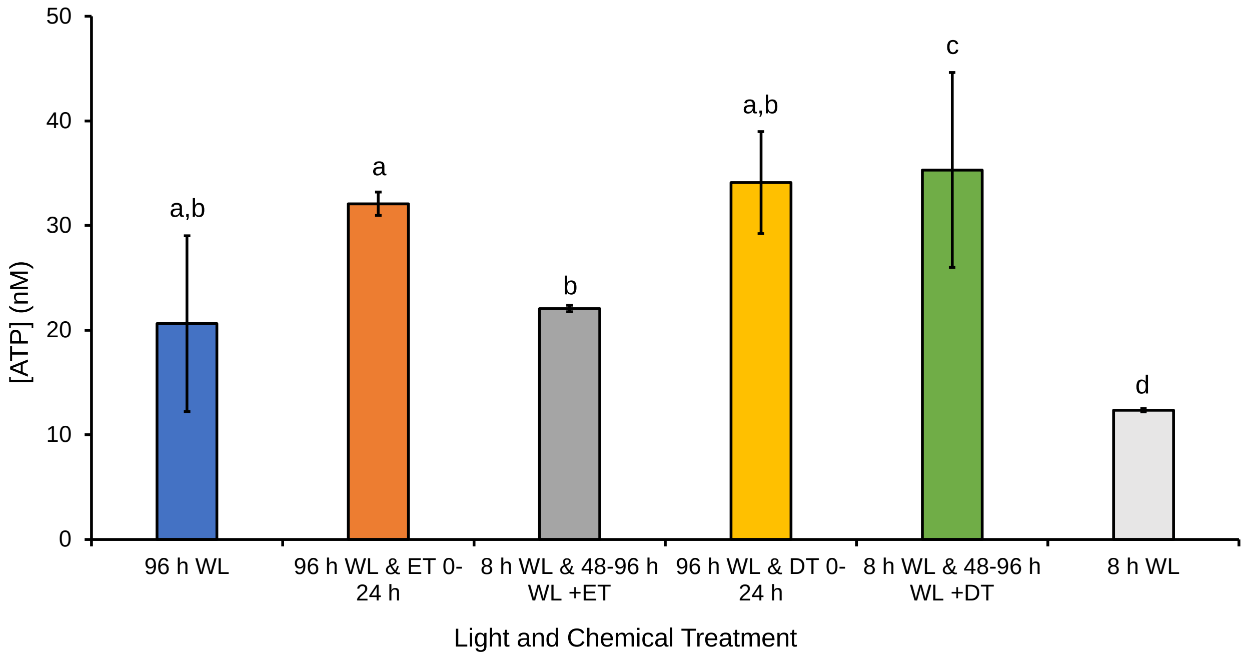

Supplemental Figure 4: Effects of light and chemical treatments on quantity of ATP extracted from spores. For all conditions with the same letter, the difference in ATP quantity is not statistically significant. If two conditions have different letters, then the quantity of ATP is statistically different.
